# Clinical Profile of Hyper-IgE Syndrome in India

**DOI:** 10.3389/fimmu.2021.626593

**Published:** 2021-02-26

**Authors:** Biman Saikia, Amit Rawat, Ranjana W. Minz, Deepti Suri, Vignesh Pandiarajan, Ankur Jindal, Smrity Sahu, Adil Karim, Mukesh Desai, Prasad D. Taur, Ambreen Pandrowala, Vijaya Gowri, Manisha Madkaikar, Aparna Dalvi, Reetika Mallik Yadav, Harsha Prasada Lashkari, Revathi Raj, Ramya Uppuluri, Venkateswaran V. Swaminathan, Sagar Bhattad, Gladys Cyril, Harish Kumar, Anuj Shukla, Manas Kalra, Geeta Govindaraj, Surjit Singh

**Affiliations:** ^1^Department of Immunopathology, Postgraduate Institute of Medical Education and Research, Chandigarh, India; ^2^Department of Pediatrics, Advanced Pediatric Centre, Postgraduate Institute of Medical Education and Research, Chandigarh, India; ^3^Department of Immunology, BJ Wadia Hospital for Children, Mumbai, India; ^4^ICMR-National Institute of Immunohaematology, Mumbai, India; ^5^Kasturba Medical College, Mangalore, India; ^6^Department of Pediatric Hematology, Oncology, Blood and Marrow Transplantation, Apollo Hospitals, Chennai, India; ^7^Aster CMI Hospital, Bangalore, India; ^8^Niruj Rheumatology Clinic, Ahmedabad, India; ^9^Sir Ganga Ram Hospital, New Delhi, India; ^10^Government Medical College, Kozhikode, Calicut, India

**Keywords:** hyper-IgE syndrome, India, STAT3 LOF, multi-centric study, rare variants

## Abstract

**Introduction:** Hyper-IgE Syndrome (HIES) is a rare inborn error of immunity (IEI) characterized by a constellation of symptoms related to susceptibility to *Staphylococcal* skin and pulmonary infections, eczema, raised serum IgE (>2,000 IU/ml), craniofacial anomalies, and recurrent bone fractures. Data on HIES from the Indian subcontinent is scarce and restricted to small case series and case reports. This is the first compilation of national data on HIES.

**Materials and Methods:** A total 103 cases clinically diagnosed and treated as HIES were analyzed from nine centers. Cases with clinical and/or molecular diagnosis of DOCK8 deficiency were not included. Patients were divided into two groups: group I for whom a heterozygous rare variant of STAT3 was identified, and group II, with clinical features similar to those of AD STAT3 deficiency, but without any genetic diagnosis.

**Results:** Genetic diagnosis was available in 27 patients (26.2%) and all harbored rare variants in the STAT3 gene. Majority of these STAT3 HIES patients presented with recurrent skin abscesses (77.7%) or pneumonia (62.9%) or both (59.2%). Other features included eczema (37%), candidiasis (55.5%), facial dysmorphism (55.5%), recurrent fractures (11.1%), and retained primary teeth (7.4%). *Mycobacterial* infections were seen in a significant 18.5%. Mortality was seen in three subjects (11.1%). A similar trend in the clinical presentation was observed when all the 103 patients were analyzed together. Twenty percent of patients without a rare variant in the STAT3 gene had an NIH score of ≥40, whereas, 51.9% of STAT3 HIES subjects had scores below the cut off of ≥40. TH17 cell numbers were low in 10/11 (90.9%) STAT3 HIES tested. Rare variants observed were 8 in exon 21; 8 in exon 13; 3 in exon 10; 2 in exon 15, and one each in exon 6, 16, 17, 19, 22, and splice site downstream of exon 12. Seven variants were novel and included F174S, N567D, L404Sfs^*^8, G419 =, M329K, T714I, R518X, and a splice site variant downstream of exon 12.

**Conclusions:** The report includes seven novel STAT3 variants, including a rare linker domain nonsense variant and a CC domain variant. *Mycobacterial* diseases were more frequent, compared to western literature.

## Introduction

Hyper-IgE Syndrome (HIES) represents a heterogenous group of disorders majorly resulting from impaired STAT3 signaling. Characteristic features include *staphylococcal* “cold” skin abscesses, *staphylococcal* pneumonia with *pneumatocele* formation, early onset eczema, muco-cutaneous candidiasis, retained primary dentition, recurrent fractures, osteoporosis, and a raised serum IgE (>2,000 IU/L). Dominant negative heterozygous STAT3 loss-of-function (LOF) mutations accounts for majority of the autosomal dominant (AD) and sporadic forms of HIES. (1, 2). Lack of TH17 cells, resulting from a defective STAT3 signaling probably accounts for only a minor fraction of the HIES disease spectrum because, inborn errors of immunity involving the IL-17 axis results in isolated chronic mucocutaneous candidiasis without any other features of HIES (3). Another feature that has emerged over the recent years is the presence of cranio-facial and dental anomalies in defects involving IL-6ST (gp130) (4–6) apart from STAT3 (1, 2). Occurrence of similar cranio-facial anomalies in IL-11R deficiency (7), that functions upstream of STAT3 through the common gp130 receptor chain (7, 8) but lack of the same in IL-6R deficiency (9) points toward a defect in IL-11/STAT3 mediated signaling as the cause for the craniofacial anomalies. While ZNF341 is required for transcription of STAT3 (10, 11), ERBB21P functions through formation of Stat3/erbin/Smad2/3 complex (12). Other molecules like PGM3 (13), and CARD11 (14) are not etiologies of bonafide HIES as they lack many features of typical HIES (15).

Diagnosis of HIES, like many other IEIs is a combination of clinical and laboratory parameters, and there is no single specific test which clinches the diagnosis. Whereas absolute eosinophil count (AEC) and serum IgE levels can be part of routine laboratory testing, TH17 cell numbers, pSTAT3 assay, and memory B cell numbers are tests that can be done only in specialized laboratories. Molecular diagnosis hence becomes imperative for clinching the diagnosis. With the advent of commercially available NGS platforms providing molecular diagnosis at a reasonable cost, it has become possible for clinicians to make a diagnosis in a suspected case even without the specialized laboratory tests. These functional tests are however still relevant even if a reverse diagnostic approach starting with NGS is considered.

The first case of HIES from India was reported in 1994 by Pherwani et al. (16). Salaria et al. (17, 18) reported three cases in two separate reports in 1997 and 2001. Pherwani and Madnani (19) reported six patients with prominent cutaneous and respiratory features, but only one had familial involvement. Patel et al. (20) reported 10 cases in 2018 but all these reports were without a molecular diagnosis. The first series of six patients of STAT3 LOF HIES with a documented genetic defect was reported by Saikia et al. (21) in 2014, that included a novel variant. This was followed by another case report with a novel variant in 2017 (22). Publications in the form of original research papers followed from the center at Chandigarh subsequently (23, 24). More research is currently being undertaken at this center with funding from Indian Council of Medical Research, New Delhi, and Jeffrey Modell Foundation, USA, but there has been a stark silence from any other center in the country except for a case report by Govindaraj et al. (25) in 2018. In a report by Gupta et al. (26) in 2012, HIES accounted for 4.9 and 16.3% of all IEIs diagnosed at two major centers in India. This manuscript is the first effort to compile data on Hyper-IgE Syndrome at a national level. Cases reported in references 19, 20, and 23 (*n* = 6) are included in this report.

## Materials and Methods

### Patients and Clinical Presentation

The cohort consisted of a total of 103 subjects. Data was compiled from from all regional centers supported by the Foundation for Primary Immunodeficiency Diseases (FPID), USA, and other centers (federal government run as well as from the private sector) providing clinical care to patients with IEIs using a common proforma that was circulated by email. These nine centers included Postgraduate Institute of Medical Education and Research, Chandigarh (number of cases contributed, *n* = 34); BJ Wadia Hospital for Children, Mumbai (*n* = 26); Indian Council of Medical Research-National Institute of Immunohaematology (ICMR-NIIH), Mumbai (*n* = 15); Kasturba Medical College, Mangalore (*n* = 13); Department of pediatric hematology, oncology, blood and marrow transplantation, Apollo hospitals, Chennai (*n* = 5); Aster CMI Hospital, Bangalore (*n* = 5); Niruj Rheumatology Clinic, Ahmedabad (*n* = 2); Sir Ganga Ram Hospital, New Delhi (*n* = 2); and Government Medical College, Kozhikode, Calicut, Kerala (*n* = 1). Cases with a clinical diagnosis of HIES with or without a molecular diagnosis and under treatment/follow-up in these centers were recruited. The following information was obtained from each participating center: age at presentation of index patient, gender, highest serum IgE levels, highest absolute eosinophil count (AEC), NIH score, family history, skin infection (with pathogen Isolates), pulmonary infections (pneumonia) with or without pneumatocele, associated TB if present, fungal infections, facies, connective tissue, and skeletal abnormalities (retention of primary teeth, minor trauma fractures, osteopenia, scoliosis, hyperextensible joints), vascular abnormalities (aneurysms, dilation of arteries), associated autoimmunity/malignancy, pSTAT3 (%), Th17 cells (%), Memory B cells (%), treatment and follow up, gene variant: gene, exon, nucleotide change, amino acid change, ACMG Classification, and whether a known or a novel rare variant. Cases with a suspected and/or molecular diagnosis of DOCK8 deficiency were not included in the study.

### Immunologic Investigations

TH17 cell enumeration, Memory B cell numbers, and pSTAT3 assay is available only at PGI Chandigarh, and were hence done in the cases that were assessed here. Five cases were however evaluated on transported samples from various centers.

### TH17 Cell Enumeration

PBMCs were isolated with Ficoll-Hypaque density centrifugation (Sigma Aldrich, St Louis, Mo). T_H_17 cells were identified by means of intracellular staining of CD4^+^ T cells for the production of IL-17. Briefly, 1 × 10^6^ cells from patients and an age matched healthy control subject were stimulated for 6 h with 10 ng/ml phorbol 12-myristate 13-acetate and 1 ug/ml ionomycin (Sigma-Aldrich, St Louis, Mo) in the presence of GolgiPlug (BD Biosciences, San Jose, CA). After cell-surface staining with PerCP-conjugated anti-CD4 (BD Biosciences, San Jose, CA), cells were fixed, permeabilized (Cytofix/Cytoperm, BD Biosciences, San Jose, CA), and stained with Alexa Fluor 647-conjugated anti-IL-17A (BD Biosciences, San Jose, CA). Immunoglobulin isotype control was used as a background control. CD4^+^ T cells were also evaluated for IFN-γ production (FITC-conjugated anti-IFN-γ; BD Biosciences, San Jose, CA). CD4^+^IL17^+^IFN-γ^−^ cells were taken as TH17 cells (Supplementary Figure 1A).

### Phospho-STAT3 Assay

One-hundred microliters of fresh whole blood were incubated with IL-6 for 15 min. Cells were simultaneously fixed and RBCs lysed using BD fix and lysing solution (BD Biosciences, USA). Cells were then permeabilized for 20 min using Perm III solution (BD Biosciences, USA) and subsequently incubated with Alexa Fluor 647 phospho-STAT3 antibody against phospho-Y705 (BD Biosciences, USA) for 30 min at room temperature. After washing twice with stain buffer, cells were suspended in 1% paraformaldehyde for acquisition (Supplementary Figure 1B).

### Memory B Cells

Memory B cells were assessed as CD19+ CD27+ cells using CD19-FITC and CD-27 APC antibodies (BD Biosciences) using standard surface staining protocols. All flow cytometry assays were performed on a BD LSR Fortessa instrument (BD Biosciences) and analyzed with Cell Quest Pro software (BD Biosciences).

### Molecular Analysis

Molecular analysis was performed either by NGS or Sanger sequencing as per availability at the referral centers. The NGS panels included a limited 44 gene panel and a 320 gene panel. The former included STAT3 and DOCK8 as the HIES associated genes while the latter included STAT3, DOCK8, TYK2, and CARD11. Sanger sequencing for the STAT3 gene was performed using a set of previously published primers. The sequencing data were analyzed using Codon Code Aligner software. Polymorphism Phenotyping program (*PolyPhen*, http://genetics.bwh.harvard.edu/pph) and Combined Annotation Dependent Deletion (*CADD*, http://cadd.gs.washington.edu) programs were used to predict the effect of the identified STAT3 rare variants.

### Statistical Analysis

Descriptive methods of statistical analysis were used using Statistical Package for Social Sciences (SPSS Inc., Chicago, IL, version 15.0 for Windows).

## Results

Of the 103 individuals, 76 were males and 27 were females, with age at diagnosis ranging from 6 months to 35 years (mean age 7.4 years, median 5 years). For analysis, patients were divided into two groups: patients of group I, for whom a heterozygous rare variant of STAT3 has been identified (*n* = 27), and patients of group II, with clinical features similar to those of patients with AD STAT3 deficiency, but without any genetic diagnosis (*n* = 76).

### Group I, HIES With Rare Variants in STAT3 Gene

Genetic diagnosis was available in 27 patients (26.2%): 19 males and 8 females from 25 kindreds with a mean age at diagnosis of 9.8 years (range 8 months to 35 years). A positive family history in the form of sib death or sibling with similar symptoms was elicited in six kindreds of which two kindreds had a clear cut autosomal dominant pattern of inheritance with an affected parent. Serum IgE levels (available in 26 subjects) of >2,000 IU/mL was observed in 19 (73%), whereas it was in the range of 1,000–2,000 IU/mL in three subjects (11.5%). Blood eosinophilia of ≥700/Cumm was seen in 63.6%. An NIH score of ≥40 was present in 13 (48.1%), and more importantly, the rest (51.9%) had a score between 20 and 39, which was below the cut-off of ≥40. Majority of the patients presented with recurrent skin abscesses (21/27, 77.7%). Pneumonia was seen in 17 (62.9%) of which *pneumatoceles* were seen in 7 (25.9%), (pyo)-pneumothorax in 4 (14.8%), and empyema in 3 (11.1%). Pneumonia with recurrent skin abscesses was seen in 16 subjects (59.2%). Other atypical sites included psoas abscess (*n* = 2), sternoclavicular abscess (*n* = 1), and orbit (*n* = 1). Eczema was seen in 10 patients (37%). Connective tissue and skeletal abnormalities were observed as follows: facial dysmorphism 15 (55.5%), hyperextensible joints 6 (22.2%), recurrent fractures 3 (11.1%), retained primary teeth 2 (7.4%), and scoliosis 2 (7.4%).

The commonest pathogen encountered was *Staphylococcus aureus* (20/27, 74%). Associated candidiasis was seen in 15 (55.5%) of which, majority were oral (*n* = 12), followed by nail (*n* = 3), lungs (*n* = 3), and skin (*n* = 1). A combination of oral candidiasis and onychomycosis was seen in two subjects. One patient had a mediastinal mass due to *Aspergillus Niger*. Other notable pathogens isolated included *Mycobacterium Abscessus complex* (*n* = 1), injection site BCG infection (*n* = 2), and *Mycobacterium tuberculosis* (MTB; *n* = 2): a case each of Pott's spine and a subcutaneous abscess in the arm, the latter showing presence of acid-fast bacilli on pus aspirate. Probable autoimmunity was encountered in 1 patient who developed anal and oral ulcerations with evidence of immune complex vasculitis on biopsy. One case of early CMV pneumonia at 2 months of age was encountered.

Absolute lymphocyte count values were available in 13 subjects and were all within normal range. CD4^+^IL17^+^ TH17 cell numbers were performed in 11 subjects and was found to be low (<0.5%) in 10 (90.9%). pSTAT3 was performed in eight subjects and was low in 3 (37.5%). Memory B cells were done in five subjects and were low in all.

**Figure 1:**
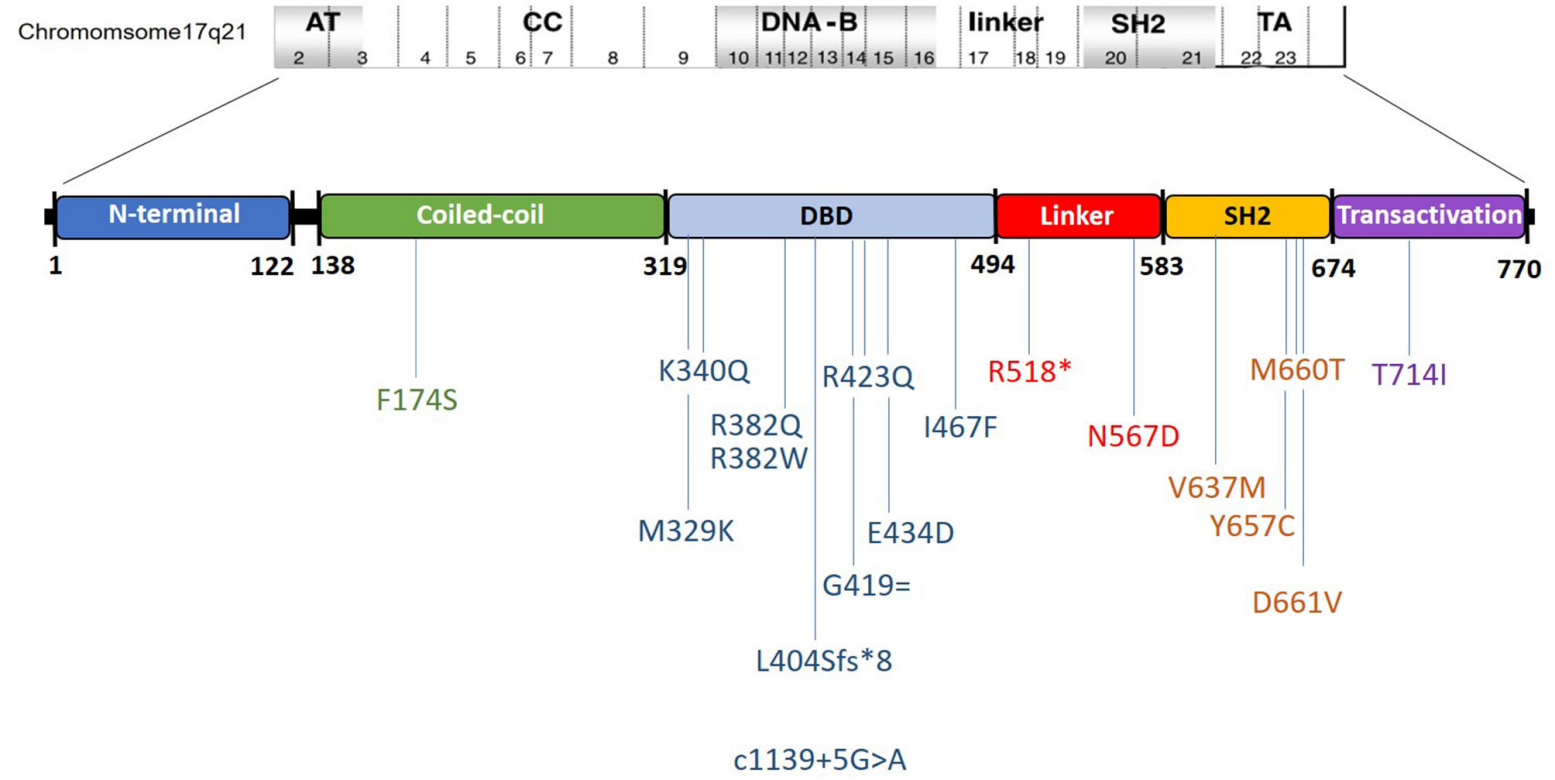
Schematic representation of the observed mutations and their location in the STAT3 gene in the present cohort. Ordinal position of nucleotide in the exon–intron boundaries according to https://www.ncbi.nlm.nih.gov/nuccore/NG_007370.1.

All 27 patients harbored rare variants in the STAT3 gene: eight in exon 21; eight in exon 13; three in exon 10; two in exon 15, and one each in exon 6, 16, 17, 19, 22, and splice site downstream of exon 12 (Figure 1, Supplementary Table 1). Of these, three were picked up by whole exome sequencing, 16 by the 320 gene NGS panel, one by the limited 44 gene NGS panel, and seven by Sanger sequencing for STAT3 gene (Supplementary Table 2). Majority were missense variants (*n* = 23), and one each of frameshift, nonsense, synonymous, and splice site rare variants. A total of 18 rare variants were identified from 25 kindreds, of which two kindreds were AD with an affected parent harboring the same STAT3 variant. Ten variants were in the DNA binding domain (DBD), four in the SH2 domain, two in the linker domain (LD), and one each in the trans-activation (TA) and coiled-coil (CC) domains (Figure 1). Seven rare variants were novel and they included F174S, N567D, L404Sfs^*^8, G419 =, M329K, T714I, R518X, and a splice site variant downstream of exon 12. The latter alteration was predicted to result in a broken WT Donor Site alteration, most probably affecting splicing and categorized as disease causing [(HSF Donor site (matrix GT) chr17:42329749 AGGGTAAGT>AGGGTAAAT 93.76>84.19 (−10.21%); MaxEnt Donor site chr17:42329749 AGGGTAAGT>AGGGTAAAT 10.45>5.83 (−44.21%)].

There were two kindreds with familial AD LOF-STAT3-HIES. One (*kindred 2*, Supplementary Table 1) was an 8 months old female child presenting with recurrent oral thrush with history of CMV pneumonia at 2 months of age and had a raised serum IgE of 1,200 IU/mL. Genetic analysis revealed a known pathogenic heterozygous variant p.V637M in exon 21 of the STAT3 gene. The same variant was found in the mother. The mother was however relatively asymptomatic and gave history only of occasional pyoderma in childhood. The second kindred (*kindred 16*, Supplementary Table 1) were a family where a 2 months old child presented to the hospital with severe pneumonia, pneumothorax, pyoderma, had typical facies and serum IgE of 2,449 IU/mL. The child however died before he could be investigated further. The father of the child, 35 years of age, had coarse facies and gave history of recurrent pneumonias since childhood. Genetic analysis of the father revealed a known pathogenic heterozygous variant p.K340Q in exon 10 of STAT3. The variant was found in another son, 5 years of age, who had recurrent upper respiratory infections and itchy skin lesions. This family has been previously reported (21). They were however lost to follow-up.

p.F174S (*kindred 1*, Supplementary Table 1) was a CC domain heterozygous rare variant in a 15 years old male with history of atopic eczema in childhood, allergic rhinitis and cellulitis in the cheek at 13 years of age. He had oral candidiasis and hyperextensible joints but his serum IgE was not raised. He developed mediastinal and abdominal lymphadenopathy with splenomegaly and abdominal lymph node biopsy showed *Mycobacterium abscessus complex*. A possibility of Mendelian Susceptibility to Mycobacterial Disease (MSMD) was considered, but no variants were found in the MSMD related genes (Supplementary Data: Case Report 1).

R518X (*kindred 20*, Supplementary Table 1) was a *de novo* heterozygous rare variant detected in a 2 years old boy presenting with pneumonia and extremely high absolute eosinophil count (21,432/Cumm). The variant was predicted to result in a truncated STAT3 protein lacking both the SH2 (required for dimerization) and the transactivation domain (containing the Y705 phosphorylation). Both the parents, and the 3 siblings were STAT3 wild type (Supplementary Data: Case Report 2).

#### Follow-Up and Treatment

Follow up data was available in 23 subjects and 4 were lost to follow up. Majority of the patients were on antimicrobial prophylaxis (*n* = 15) and doing well on follow-up. IVIG was instituted in 3 subjects. The patient with p.F174S variant had an associated Klinefelter's Syndrome (46XXY). The patient with p.T714I variant developed a refractory *E coli* psoas abscess that required multiple surgical drainage. Patient with pR518X variant developed pulmonary symptoms for which a bronchoalveolar lavage was done that showed plenty of eosinophils and diagnosis of eosinophilic pneumonitis was considered and the patient put on oral steroids to which he responded. Mortality was observed in three male subjects (11.1%) aged 2, 4, and 15 years, all due to pneumonia and related complications. One patient with pD661V variant had a peripheral T cell Lymphoma and underwent a matched unrelated HSCT at Apollo Hospital, Chennai and is currently in remission, 3 months post HSCT.

### Group II, HIES Without Documented Genetic Diagnosis

There were 76 patients where a clinical suspicion of HIES was entertained but a rare variant could not be demonstrated on genetic analysis or where genetic studies was not done. Genetic analysis was attempted in 30 subjects: 25 by sanger sequencing for STAT3 gene, four using the limited 44 gene NGS panel, and one by whole exome sequencing. The limited 44 gene NGS panel however did not contain the more recently described genes associated with HIES viz. IL-6R, IL-6ST, ZNF341, PGM3, CARD11, and ERBB21P.

There were 57 males and 19 females with age at diagnosis ranging from 6 months to 27 years (mean 6.5 years). A positive family history was elicited in 16 (21%). Serum IgE levels of >2,000 IU/mL was seen in 61/75 (81.3%), whereas it was between 1,000 and 2,000 IU/mL in 5/75 (6.6%). Blood eosinophilia of ≥700/Cumm was found in 39/68 (57.3%). Eczema was seen in 44 (57.8%). An NIH score of ≥40 was encountered in 20 (26.3%) and in majority (53; 69.7%), the score ranged from 20 to 39. Connective tissue and skeletal anomalies recorded included facial dysmorphism in 50 (65.7%), hyper-extensible joints 15 (19.7%), high arched palate in 4 (5.2%), retained primary teeth in 2 (2.6%), recurrent fractures in 2 (2.6%), and scoliosis in 2 (2.6%).

Majority presented with history of recurrent skin abscesses (46; 60.5%) and/or pneumonia (45; 59.2%), both being present in 43 (56.5%). Pulmonary complications were seen as follows: pneumatocele in 6 (7.8%), bronchiectasis in 3 (3.9%), and pneumothorax in 2 (2.6%). One patient had associated psoriasis. Other manifestations included recurrent diarrhea, pyomyositis, renal abscess, and liver abscess (one case each).

Pathogen isolates included *S. aureus* (48; 63.1%) and one case each of S*treptococcus pyogenes, Pseudomonas* spp., *Acenatobacter*, and *E. coli*. Candidal infections were seen in 18 subjects (23.6%) and included 15 oral, three nail, and one case each of gastro-intestinal, skin and lung candidiasis. *Aspergillus* sinusitis was seen in one case. *Mycobacterial* infections were seen in 5: pulmonary MTB infection in 4 and local site BCG infection in 1. TH17 cells were examined in 19 cases and were found to be low in 11 (57.8%). pSTAT3 assay was done in 21 subjects and was low in 9 (42.8%).

#### Follow-Up and Treatment

Follow-up data were available in 52 subjects, and 24 were lost to follow-up. Of those with follow-up, 49 were on antimicrobial prophylaxis and doing well. Three patients, 2 females and a male aged 6 months, 1 year and 8 years died due to pneumonia and related complications.

Clinical features of the STAT3 HIES group, the group without a genetic diagnosis and the entire cohort combined is summarized in Table 1.

**Table 1:** Summary of clinical features.

**Clinical feature**	**HIES with mutation in STAT3**		**HIES without mutation demonstrated**		**HIES, all**	
**Mean age at diagnosis**	9.8 years		6.5 years		7.4 years	
	**Number**	**%**	**Number**	**%**	**Number**	**%**
NIH score ≥ 40	13/27	48.1	20/76	26.3	33/103	32.0
Serum IgE ≥ 2,000 IU/mL	19/26	73	61/75	81.3	80/101	79.2
Eosinophilia ≥ 700/Cumm	14/22	63.6	39/68	57.3	53/90	58.8
Recurrent skin abscesses	21/27	77.7	46/76	60.5	67/103	65.0
Recurrent pneumonia	17/27	62.9	45/76	57.8	62/103	60.1
Pneumatoceles	7/27	25.9	6/76	7.8	13/103	12.6
Eczema	10/27	37%	44/76	57.8	54/103	52.4
Facial dysmorphism	15/27	55.5	50/76	65.7	65/103	63.1
Hyper-extensible joints	6/27	22.2	15/76	19.7	21/103	20.3
Retained primary teeth	2/27	7.4	2/76	2.6	4/103	3.8
Recurrent fractures	3/27	11.1	2/76	2.6	5/103	4.8
Staphylococcal infection	20/27	74	48/76	63.1	68/103	66.0
Candidiasis	15/27	55.5	18/76	23.6	33/103	32.0
Mycobacterial infections	5/27	18.5	5/76	6.5	10/103	9.7
Autoimmunity	1/27	3.7	1/76	1.3	2/103	1.9
Malignancy	1/27	3.7	–	–	1/103	0.9
Mortality	3/23	13	3/52	5.7	6/75	8.0

## Discussion

Though HIES has been recognized and reported from India as early as 1994, when genetic cause of the disease was still unknown, 103 cases compiled from the entire country with a population of 1,366 million (2019 census) clearly indicates it is an under recognized and under reported entity. With organizations like FPID and Indian Society for Primary Immune Deficiency (ISPID) involved in awareness campaigns amongst the medical fraternity for nearly the entire last decade, cases are now being recognized more often. With availability of commercial as well as federal government run NGS facilities, clinicians are now at a position to get a molecular diagnosis even without resorting to functional assays. The ICMR advanced center for diagnosis of PID at PGIMER, Chandigarh has taken forefront in diagnosis and research in HIES and has been providing services for assays like TH17 and pSTAT3. However, conducting these assays on transported samples from distant centers under hot and humid conditions prevailing through major part of the year has been largely frustrating.

This cohort of patients did not include patients with DOCK8 mutations in keeping with the fact that DOCK8 deficiency is considered a combined immunodeficiency and hence classified therein (27). Since STAT3 deficiency accounts for more than 90% of all autosomal dominant and sporadic forms of HIES, it's over representation with only STAT3 defect in the current cohort is understandable. However, non-representation of other genetic variants associated with HIES could be because of the lack of genetic testing in majority, and even in those who were tested, the gene panels employed did not contain the relevant genes and hence were likely to be missed.

We compared the clinical profile of our cohort to two published large cohorts: the USIDNET (28) and the French cohort (29). While the former was a clinical cohort of 85 patients without reference to their gene variants, and hence a heterogenous group with or without genetic diagnosis, the French cohort was a cohort of 67 patients from 47 kindreds with exclusively the STAT3 defect, autosomal dominant as well as sporadic. For the sake of uniformity, we compared our cases with a rare variant in the STAT3 gene with the French cohort and then our entire cohort with the USIDNET cohort. Two Chinese cohorts (30, 31) were included as Asian cohorts for comparison.

Majority of our patients with STAT3 rare variants presented with recurrent skin abscesses (77.7%) and pneumonia (62.9%) which were seen in 73 and 38% of the French cohort. Early onset eczema was 37% in our STAT3 HIES cohort but was observed in larger numbers the French cohort (48%). *S. aureus* was the commonest pathogen isolated (74%) which was seen in 94% of the French cohort. Candida was seen in 55.5% of our patients, which was 85% of the French cohort. Though majority of the patients without a genetic diagnosis had an NIH score between 20 and 39, 20% had a score ≥40. This was seen in 56% of the cases of suspected HIES without a STAT3 rare variant in the study by Woellner et al. (32). On the other hand, majority (51.9%) of our patients with a documented STAT3 rare variant had scores below cut off of ≥40.

*Tuberculosis* is an endemic disease in India and accounts for more than 27% of tuberculosis worldwide [*Global Tuberculosis report 2019. World Health Organization (WHO), P1,2*]. Hence, unlike western literature, presence of TB in the pathogen spectrum of HIES in India is not unexpected. Similar to other Asian cohorts, in our cohort, TB and related mycobacterial infections was seen in 18.5% of the STAT3 cohort. This included MTB as well as atypical mycobacteria (*Mycobacterium abscessus complex* and *BCG*). In two different Chinese cohorts (30, 31), 37.5 and 38.8% incidence of BCG complications have been reported that included local BCG site abscess/ulceration as well as disseminated *BCGosis*. BCG related complications were observed in 2 of our STAT3 HIES patients. *Mycobacterial* infections in HIES have been observed in other studies as well (33, 34). Malignancy in the form of a Non-Hodgkins' Lymphoma (NHL) was observed in one of our STAT3 HIES patients (0.9%). Malignancy has been observed in 7% of the patients in the French cohort all of which were NHL.

Comparison of our entire cohort of 103 patients with the USIDNET cohort showed similar trends: Staphylococcal skin abscesses 65 vs. 74.4%; pneumonia 60.1 vs. 72%; eczema 52.4 vs. 57.3%. *S. aureus* was the commonest organism isolated (66 vs. 72.3%), followed by candida (32 vs. 25.9%). Molluscum was seen in five of our 103 patients which was seen in four patients in the USIDNET cohort. Mycobacterial infections accounted for 9.7% when the entire cohort was considered.

Majority of the reported STAT3 rare variants are described in the DBD and SH2 domains which are known mutation hotspots in the STAT3 gene (1, 2, 29). DBD and SH2 domain rare variants together comprised 77.8% of the variants in our present cohort. Rare variants were however observed through all the five domains in the present cohort, including the LD and CC domain. R382Q/W was the commonest variant seen in our cohort, comprising 7/27 patients (25.9%) followed by V637M, seen in 4/27 patients (14.8%) and F174S, seen in 2/27 patients (7.4%). R382Q/W accounted for 34% of the rare variants in the French kindreds and 22.8 and 45% of the Chinese cohorts. V637M similarly accounted for 17.4% in the Chinese and 10.6% in the French cohort.

STAT3 Linker domain mutations are rare (35, 36) and constitute <2% in the larger series (1, 2, 29, 37) and few publications as case reports are found in the literature (35). Majority of reported variants in STAT3 are missense variety, and non-sense variants in the STAT3 gene are not frequent (1, 2, 29, 37). This made the R518X variant in our cohort an extremely rare variant; a null variant in the linker domain of STAT3. As the predicted truncated STAT3 protein lacking both the SH2 and TA domains was not expected to exert a dominant negative effect, haploinsufficiency (HI) as a possible disease mechanism was contemplated. This would however need functional assessment of the rare variant which is being currently carried out. HI as a disease mechanism has been proposed previously by Natarajan et al. (38) in a c.1140-3C>G; p.S381^*^ null variant. Mutations in the CC domain of STAT3 hasn't yet been reported in literature and the p.F174S in our cohort hence is an extremely rare variant.

Cranio-facial, dental, and skeletal features were seen in a minority of our patients: facial dysmorphism in 55.5% of the STAT3 and 63.1% of the entire cohort, retained primary teeth in 7.4 and 3.8%, and recurrent fractures 11.1 and 4.8%. While delayed shedding and retained primary dentition indicates decreased osteoclast (OC) function, recurrent fractures and osteoporosis denotes increased OC activity, which are contradictory. Studies conducted at the Chandigarh center to look at the pathogenesis of cranio-facial and dental manifestations in HIES by looking at genes involved in bone homeostasis revealed *osteopontin* (OPN) as a candidate gene that was altered significantly in patients with HIES and STAT3 deficient cell lines (24). The OPN gene was also shown to have hitherto undescribed STAT3 response elements in its promoter region by *in silico* studies (24). Interestingly, differential expression of OPN was observed in patients with HIES even before the STAT3 era (39). Though patients with STAT3 rare variants do not show obvious alterations in OC morphology, differential expression of genes like NFaTc1, STAT3, and OPN has been observed in OCs from HIES subjects and mutant cell lines (unpublished observations).

pSTAT3 assay by flowcytometry is considered to be an important functional analysis in STAT3 LOF HIES and assesses the canonical STAT3 pathway mediated through pY705 phosphorylation in the TA domain. The assay however can be normal in a significant majority and hence a normal pSTAT3 expression doesn't rule out the disease. pSTAT3 was done in 8 of the 27 STAT3 HIES patients in our cohort and was normal in a majority (62.5%). The non-canonical pathway of STAT3, that do not require pY705 phosphorylation (unphosphorylated STAT3, uSTAT3) has been shown to act through NFkB mediated RANTES, IL-8 and other IFN-γ response elements (40, 41). Investigation showed a downregulation of RANTES, IL-8, and IFNβ genes in patients with HIES (unpublished observations) which could further contribute to the immune deficiency in these patients.

## Conclusions

We report here a multi centric cohort of 103 HIES patients from India, of which 27 were STAT3 HIES. Though molecular diagnosis was available only in 27 patients, the 18 STAT3 variants detected included seven novel rare variants, including a rare LD nonsense variant and a CC domain variant. *Mycobacterial* diseases were more frequent, similar to other Asian cohorts, and hence need to be considered in the pathogen spectrum of HIES in India in addition to the usual *Staphylococcus* and *candida* infections. Notably, more than half of our STAT3 HIES subjects had low NIH scores, that was below the cut off of ≥40. With increasing awareness and better availability of molecular diagnostic facilities, more and more cases of HIES are likely to be reported and diagnosed.

## Data Availability Statement

The raw data supporting the conclusions of this article will be made available by the authors, without undue reservation.

## Ethics Statement

The studies involving human participants were reviewed and approved by Institute Ethics Committee, PGIMER, Chandigarh. Written informed consent to participate in this study was provided by the participants' legal guardian/next of kin.

## Author Contributions

BS collected the data, did the analysis, and wrote the paper. SmS, AK, and AD did the flowcytometry experiments and analyzed the genetic analysis data. SuS, DS, AR, MD, PT, AP, VG, MM, AD, RM, HL, RR, RU, VV, SB, GC, HK, AS, MK, and GG provided patient data and conducted clinical exploration and treatment of the subjects. RM, AR, DS, VP, and SuS did a critical review of the manuscript. All authors contributed to the article and approved the submitted version.

## Conflict of Interest

The authors declare that the research was conducted in the absence of any commercial or financial relationships that could be construed as a potential conflict of interest.

## Supplementary Material

Supplementary Figure 1 **(A)** Representative flow cytometry contour plots for TH17 (CD4^+^IL17^+^IFN-γ^−^) cell estimation. Upper panel is a healthy control's plot (TH17 cells = 2.4%) and lower panel from a suspected patient of HIES (TH17 cells = 0.4%). ST, Stimulated with PMA-Ionomycin; US, Unstimulated. **(B)** Representative flow cytometry histogram plots for pSTAT3 assay. Upper panel is from a healthy control sample (MFI 479) and lower panel from a HIES subject (MFI 228). ST, Stimulated with IL-6; US, Unstimulated.

Supplementary Table 1 List of STAT3 mutations with their CADD and SIFT scores.

Supplementary Table 2 List of genes in the targeted 44 gene NGS panel and the 320 gene NGS panel.
