# Supplementary figures and images for: Clinical Profile of Hyper-IgE Syndrome in India
Source: Front Immunol. 2021 Feb 26;12:626593. doi: 10.3389/fimmu.2021.626593 (PMC7952512; doi:10.3389/fimmu.2021.626593)

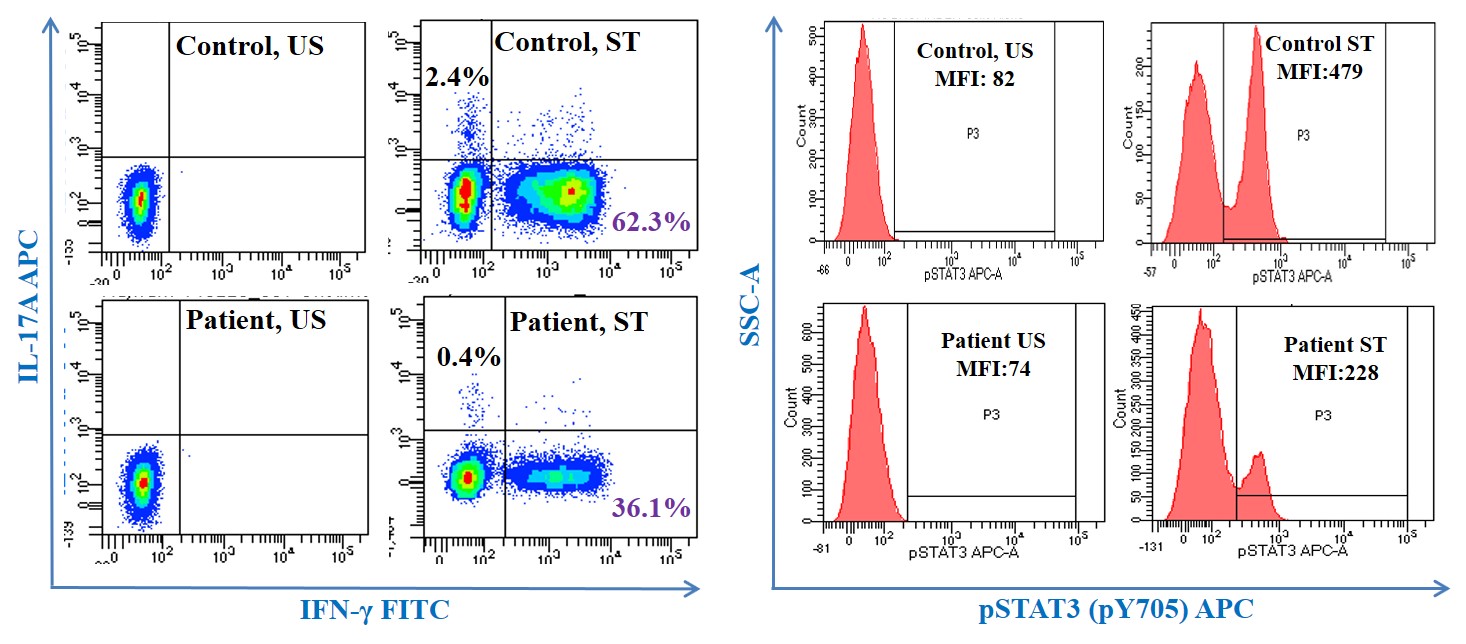

Supplement: Supplementary Figure 1 — (A) Representative flow cytometry contour plots for TH17 (CD4+IL17+IFN-γ−) cell estimation. Upper panel is a healthy control's plot (TH17 cells = 2.4%) and lower panel from a suspected patient of HIES (TH17 cells = 0.4%). ST, Stimulated with PMA-Ionomycin; US, Unstimulated. (B) Representative flow cytometry histogram plots for pSTAT3 assay. Upper panel is from a healthy control sample (MFI 479) and lower panel from a HIES subject (MFI 228). ST, Stimulated with IL-6; US, Unstimulated. [file Image_1.JPEG]
