# Supplementary material for: Clinical Profile of Hyper-IgE Syndrome in India
Source: Front Immunol. 2021 Feb 26;12:626593. doi: 10.3389/fimmu.2021.626593 (PMC7952512; doi:10.3389/fimmu.2021.626593)
Supplement: Supplementary Table 2 — List of genes in the targeted 44 gene NGS panel and the 320 gene NGS panel. [file Table_2.DOCX]

**Supplementary Table 2:**

**PID panel (44 gene)**

ADA, AICDA, AIRE, ATM, BTK, C1QA, C1QB, C1QC, CD40, CD40LG, CFH, CFP, CTLA4, CYBA, CYBB, **DOCK8**, FAS, FASLG, FOXP3, GATA2, ICOS, IFNGR1, IFNGR2, IL10RA, IL10RB, IL12RB1, IL17F, IL17RA, IL2RG, IL7R, ITGB2, JAK3, LIG4, LRBA, NCF1, NCF2, NCF4, PRF1, RAG1, RAG2, STAT1, **STAT3**, STX11, WAS

**NGS Panel (320 genes)**

ACP5, ACTB, ADA, ADAM17, ADAR, AICDA, AIRE, AK2, AP1S3, AP3B1, AP3D1, APOL1, ATM, ATP6AP1, B2M, BCL10, BCL11B, BLM, BLNK, BTK, C1QA, C1QB, C1QC, C1R, C1S, C2, C3, C5, C6, C7, C8A, C8B, C8G, C9, **CARD11**, CARD14, CARD9, CASP10, CASP8, CCBE1, CD19, CD247, CD27, CD3D, CD3E, CD3G, CD40, CD40LG, CD46, CD55, CD59, CD79A, CD79B, CD81, CD8A, CDCA7, CEBPE, CECR1, CFB, CFD, CFH, CFHR1, CFHR2, CFHR3, CFHR4, CFHR5, CFI, CFP, CFTR, CHD7, CIITA, CLCN7, CLPB, COPA, CORO1A, CR2, CSF2RA, CSF2RB, CSF3R, CTC1, CTLA4, CTPS1, CTSC, CXCR4, CYBA, CYBB, DCLRE1C, DDX58, DKC1, DNAJC21, DNMT3B, DOCK2, **DOCK8**, ELANE, EPG5, ERCC6L2, EXTL3, FAAP24, FADD, FAS, FASLG, FAT4, FCGR3A, FCN3, FERMT3, FOXN1, FOXP3, FPR1, G6PC3, G6PD, GATA2, GFI1, HAX1, HELLS, HMOX1, ICOS, IFIH1, IFNAR2, IFNGR1, IFNGR2, IGHM, IGKC, IGLL1, IKBKB, IKZF1, IL10, IL10RA, IL10RB, IL12B, IL12RB1, IL17F, IL17RA, IL17RC, IL1RN, IL21R, IL2RA, IL2RG, IL36RN, IL7R, INO80, IRAK1, IRAK4, IRF2BP2, IRF3, IRF7, IRF8, ISG15, ITCH, ITGB2, ITK, JAGN1, JAK1, JAK3, KDM6A, KMT2D, LAMTOR2, LAT, LCK, LIG1, LIG4, LPIN2, LRBA, LYST, MAGT1, MALT1, MAP3K14, MASP2, MCM4, MEFV, MKL1, MOGS, MS4A1, MSH6, MSN, MTHFD1, MVK, MYD88, MYSM1, NBAS, NBN, NCF2, NCF4, NCSTN, NDNL2, NFAT5, NFKB1, NFKB2, NFKBIA, NHEJ1, NHP2, NLRC4, NLRP1, NLRP12, NLRP3, NOD2, NOP10, OBFC1, ORAI1, OSTM1, OTULIN, PARN, PEPD, PGM3, PIK3CD, PIK3R1, PLCG2, PLEKHM1, PMS2, PNP, POLA1, POLE, POLE2, PRF1, PRKCD, PRKDC, PSEN1, PSENEN, PSMB8, PSTPIP1, PTEN, PTPRC, RAB27A, RAC2, RAG1, RAG2, RANBP2, RASGRP1, RBCK1, RFX5, RFXANK, RFXAP, RHOH, RLTPR, RMRP, RNASEH2A, RNASEH2B, RNASEH2C, RNF168, RNF31, RNU4ATAC, RORC, RPSA, RTEL1, SAMD9, SAMD9L, SAMHD1, SBDS, SEMA3E, SERPING1, SH2D1A, SH3BP2, SLC29A3, SLC35C1, SLC37A4, SLC46A1, SMARCAL1, SMARCD2, SNX10, SP110, SPINK5, STAT1, STAT2, **STAT3**, STAT5B, STIM1, STK , STX11, STXBP2, TAP1, TAP2, TAPBP, TAZ, TBK1, TBX1, TCF3, TCIRG1, TCN2, TERC, TERT, TFRC, THBD, TICAM1, TINF2, TIRAP, TLR3, TMC6, TMC8, TMEM173, TNFAIP3, TNFRSF11A, TNFRSF13B, TNFRSF13C, TNFRSF1A, TNFRSF4, TNFSF11, TNFSF12, TPP1, TPP2, TRAC, TRAF3, TRAF3IP2, TREX1, TRNT1, TTC37, TTC7A, **TYK2**, UNC13D, UNC93B1, UNG, USB1, USP18, VPS13B, VPS45, WAS, WDR1, WIPF1, WRAP53, XIAP, ZAP70, ZBTB24
