# Supplementary material for: Clinical Profile of Hyper-IgE Syndrome in India
Source: Front Immunol. 2021 Feb 26;12:626593. doi: 10.3389/fimmu.2021.626593 (PMC7952512; doi:10.3389/fimmu.2021.626593)
Supplement: Supplementary file 4 [file Data_Sheet_1.docx]

**Supplementary data**

**Case report 1.** 14-year-old male; product of nonconsanguineous marriage, presented with atopic eczema since early childhood, asthma poorly controlled on controller medications, and allergic rhinitis. He also reported recurrent oral candidiasis from the age of 10 that responded to topical antifungal therapy. At 13.5 years of age, he developed cheek cellulitis that responded to oral antibiotics. At the age of 14, he presented with a cough for 2 months. On evaluation, he was noted to have enlarged mediastinal and abdominal nodes along with splenomegaly. Abdominal LN biopsy yielded atypical mycobacteria, confirmed to be *Mycobacterium abscessus complex* by culture. Immunological workup showed normal immunoglobulins including normal IgE. IgA-88.7mg/dl, IgG-957mg/dl, IgM-161mg/dl, IgE-6.1 IU/ml. Th17 cells were markedly reduced as compared to healthy control on flow cytometry. Further, a clinical exome by NGS detected a mutation in exon-6 of STAT-3 gene -heterozygous-c.521T>C (p.F174S). The variant is likely damaging based on in silico prediction studies. No significant variants were noted in any other genes associated with MSMD or any other immune deficiency. Karyotype revealed 47XXY suggestive of *Klinefelter* *syndrome*. Patient is doing fine on anti-mycobacterial therapy for the past 1 year.

**Case report 2.** Two and half years old male child born to non-consanguineous parents with three elder siblings, 2 girls and a boy. Presented with fever for 4 days and skin abscesses over right lower chest (5×4 cms) and left calf (2×2 cms), boggy and lacked warmth and redness. He had a possible varicella infection at 1 year of age. At 2 years he was admitted for community acquired pneumonia with right pyo-pneumothorax requiring intercostal drainage and developed lower lobe lung collapse. Empyema fluid grew methicillin sensitive Staphylococcus aureus. Marked eosinophilia was noted on WBC differential count (57%) with an absolute eosinophil count of 21,432/Cumm. Serum IgE was 9752 IU/ml. His NIH score was 23. pSTAT3 was 51.5%, MFI 341 (Control 50.5% MFI 733). TH17 cells were low; 0.3% (control 0.8%). Genetic analysis showed a heterozygous, nonsense mutation in exon 17 (linker domain) of the STAT3 gene g.68737 C>T, c.1552 C>T, p.R518X. Both parents and the siblings were STAT3 wild type.
